# The strength of sensitivity to ambiguity

**DOI:** 10.1007/s11238-018-9657-9

**Published:** 2018-03-19

**Authors:** Robin Cubitt, Gijs van de Kuilen, Sujoy Mukerji

**Affiliations:** 1 School of Economics and Centre for Decision Research and Experimental Economics, University of Nottingham, Nottingham, UK; 2 Department of Economics, Tilburg University, Tilburg, The Netherlands; 3 School of Economics and Finance, Queen Mary University of London, London, UK

**Keywords:** Ambiguity sensitivity, Ambiguity attitude, Measuring strength of ambiguity sensitivity, Smooth ambiguity model, Ambiguity premium

## Abstract

We report an experiment where each subject’s ambiguity sensitivity is measured by an ambiguity premium, a concept analogous to and comparable with a risk premium. In our design, some tasks feature known objective risks and others uncertainty about which subjects have imperfect, heterogeneous, information (“ambiguous tasks”). We show how the smooth ambiguity model can be used to calculate ambiguity premia. A distinctive feature of our approach is estimation of each subject’s subjective beliefs about the uncertainty in ambiguous tasks. We find considerable heterogeneity among subjects in beliefs and ambiguity premia; and that, on average, ambiguity sensitivity is about as strong as risk sensitivity.

## Introduction

Introspection suggests that, in the real world, agents are often not certain of the probabilities of the different possible outcomes of an action. In decision theories motivated by this consideration, decision makers are said to perceive *ambiguity* if they are uncertain about the probability distribution governing the contingent states on which the consequences of given actions depend. Numerous experimental studies, largely built on Ellsberg (1961)’s classic examples, show that subjects’ behavior is often sensitive to ambiguity (for reviews see e.g. Camerer and Weber 1992; Trautmann and van de Kuilen 2016; Wakker 2010). For instance, subjects may display an ambiguity-averse attitude: intuitively put, being inclined to shade evaluations of acts whose consequences are less robust to the perceived ambiguity. In this paper, we report an experimental investigation in which we measure the *strength* of ambiguity sensitivity, and how that strength is distributed across the sampled population.

This is timely for wider developments in the economics of uncertainty because there is now a large and rapidly growing theoretical literature—surveyed, for example, in Etner et al. (2012), Gilboa and Marinacci (2013) and Mukerji and Tallon (2004)—that formalizes ambiguity sensitive preferences in different ways and applies them in economic models. Yet, the importance of theoretical demonstrations of possible economic consequences of ambiguity sensitivity presumably depends on the strength of the sensitivity. For example, even if many agents are ambiguity-averse, that fact probably would not affect their behavior much if the aversion is very weak. And, it is not just the average level of sensitivity that matters, but also the distribution. For example, if some agents are (economically) significantly ambiguity-seeking and others significantly ambiguity-averse, this would give rise to richer possibilities for trade in asset markets than if the only ambiguity sensitivity was ambiguity aversion.

For these reasons, we quantify ambiguity sensitivity at the individual level. Though we are not the first to do so, our approach is distinctive in both the measure used and the form of ambiguity considered. As we explain, our measure has a more natural economic interpretation and our experimental design captures better important features of ambiguity in the real world, namely that agents are often neither completely informed nor completely uninformed about probabilities and may differ in their information.

We measure the strength of ambiguity sensitivity by inferring, from the choices a subject makes, the *ambiguity premium* she associates with a given ambiguous act. The notion of ambiguity premium is analogous to that of risk premium. An agent’s risk premium for a lottery is its expected value minus the agent’s certainty equivalent. Equivalently, it is the certainty equivalent of a hypothetical risk neutral agent minus that of the real agent, with the hypothetical agent assumed to have the same information as the real one. Thus, intuitively, the risk premium is the amount that a (risk averse) agent shades off the valuation of a lottery because of his attitude to risk. By an analogous intuition, the ambiguity premium is the amount that an (ambiguity-averse) agent shades off the valuation of an ambiguous act because of his attitude to its perceived ambiguity. More precisely, it is a hypothetical *ambiguity-neutral* agent’s certainty equivalent for the act minus that of the real agent, with—importantly—the hypothetical agent assumed to share the actual agent’s subjective information *and* attitude to objective risk. The need to attribute the same subjective information and risk attitude to the real and hypothetical agents gives rise to key features of our design and approach.

Several reasons commend this concept of ambiguity premium as a useful measure of ambiguity sensitivity. First, an agent’s ambiguity premium is easily compared with their risk premium in terms of how much it shades off the expected value of an act, allowing the relative importance of the two attitudes to be assessed. Such assessment is important, to inform priorities in economic modelling. Second, although some model must be applied to compute the ambiguity premium, the concept of the premium makes no reference to any model, as we have just indicated, and is a monetary sum. In this respect, the measure contrasts with model specific ambiguity attitude parameters from the theoretical literature whose values are not portable across classes of preferences. For example, magnitudes of the parameter(s) governing ambiguity attitude in the smooth ambiguity model (Klibanoff et al. 2005) cannot be meaningfully compared with α in the α-MEU model (Ghirardato et al. 2004). Moreover, “more ambiguity averse”, an ordering on preferences that underlies the interpretation of ambiguity attitude parameters, requires that preferences may be so ordered only if they share the same risk attitude (Epstein 1999; Ghirardato and Marinacci 2002). Comparisons of ambiguity premia are not limited in this way. Third, as Trautmann and van de Kuilen (2016) documents, there is an extensive record of a measure that is loosely comparable to our ambiguity premium in the literature using Ellsberg-like 2-urn examples, namely the difference between the certainty equivalent of the lottery based on a draw from an urn with a known mixture of balls with the certainty equivalent of a “lottery” based on a draw from an urn with the unknown mixture. A limitation of this widely used traditional measure is that it implicitly assumes that subjects know nothing about the composition of the unknown mixture. We advance the literature by avoiding this limitation, refining the measure of ambiguity premium applied and, as explained above, by using a distinctive experimental design that allows a more realistic form of uncertainty.

Our design captures two features of real world decision making. First, just as agents are often not certain of the probability distribution over contingent states relevant to an action, so they are also often not completely uncertain of it. Second, different agents typically have different subjective information about the likelihoods of relevant contingences. Our design contrasts with a more standard approach in which all subjects are given the same, minimal, information about the contents of an “Ellsberg urn”. Our subjects do evaluate bets on draws of a ball from the equivalent of an “Ellsberg urn” containing a mixture of balls of different colours. But, each subject is given some subjective and incomplete information about the determination of that mixture. For example, a subject might have information that allows him to believe that one colour is more likely than the other but not precisely how likely. Our design embeds treatments and procedures intended specifically to promulgate heterogeneity of beliefs, because our objective is to demonstrate the feasibility of measuring ambiguity sensitivity while allowing for such heterogeneity.

The subjective, incomplete and heterogeneous nature of the information that subjects have in our design about (the analogue of) the “unknown mixture” presents a challenge as to how to impute the certainty equivalent(s) of the hypothetical ambiguity-neutral agent(s) when calculating ambiguity premium. We overcome the challenge by fitting the choice data of each subject to the smooth ambiguity model (Klibanoff et al. 2005), a preference model with the distinctive feature that it allows a parametric separation of belief, ambiguity attitude and risk attitude. Using estimates of the parameters, we construct the desired imputation for each subject and thus obtain an estimate of their ambiguity premium. The smooth ambiguity model parameterizes beliefs by incorporating a subjective *second*-*order* belief, making it a natural candidate for fitting the choice data from our experiment, as we will explain. However, the fact that a second-order belief is involved is widely regarded as making the estimation of the parameters of the smooth ambiguity model a particular challenge (see, e.g., Wakker 2010, p. 337; Carbone et al. 2017, p. 89). We are motivated, in part, by the goal of showing that a subject’s second-order belief can be estimated solely through revealed preference, that is by observing his choices over *first*-*order* acts.

Our findings suggest that our design succeeds in creating real ambiguity; that our subjects vary in their beliefs about it and also in their attitudes to it, even allowing for their different beliefs; and finally that, on average among our subjects, ambiguity aversion is about as economically significant as risk aversion.

The remainder of the paper is organized as follows: Sect. 2 discusses related literature. Section 3 describes the smooth ambiguity model and our experimental design. Section 4 presents our empirical findings and Sect. 5 concludes. Appendices A–C give more details of our experimental findings and procedures. For reasons mentioned in Sect. 3, Appendix D briefly discusses an alternative design to the one used.

## Related literature

In this section, we discuss other research whose main objective, like ours, is to measure ambiguity sensitivity.^1^ One goal at the boundary of such research is to try to identify the *existence* and *direction* of such sensitivity, by classifying individual subjects as ambiguity-neutral, ambiguity-seeking or ambiguity-averse.^2^ Here, we consider literature which goes beyond such typology by attempting to quantify the *strength* of sensitivity. Such quantifications take two main forms in the literature: estimation of parameters that govern ambiguity sensitivity in particular preference models; and calculation of measures akin to ambiguity premia. We consider these in turn.^3^

In some cases, models of ambiguity sensitive preference are estimated primarily for the purpose of comparing the empirical performance of the estimated models. Recent contributions of this kind include Conte and Hey (2013), Hey et al. (2010), Hey and Pace (2014) and Kothiyal et al. (2014). Papers that put more stress on the strength of ambiguity sensitivity indicated by the estimated parameters include Ahn et al. (2014), Gneezy et al. (2015) and Dimmock et al. (2015).

In Ahn et al.’s study, participants were endowed with a linear budget set and chose how much of their endowment to allocate between three assets—each formulated as a bet on a draw of a ball from an Ellsberg 3-colour urn. Their use of this allocation task is different from our approach, in which (as we explain below) certainty equivalents are elicited for objects more akin to Ellsberg 2-colour urns. Also, when Ahn et al. estimate the other parameters of the smooth model from their data they simply assume the second-order belief to be the uniform distribution (rather than estimate it, as we do).

Gneezy et al. (2015) estimates a CRRA risk attitude function and the parameter α in an α-MEU model using subjects’ evaluations of bets from draws from an Ellsberg 2-colour urn type situation where it is assumed that, “… each individual knew that the number of balls matching her success colour lay between 0 and 100.” Our approach also uses evaluations of bets, but is different to that of Gneezy et al. because we give subjects more information about the likelihood of different first order probabilities and because, in order to capture the subject’s response to that information, we use the smooth ambiguity model rather than the α-MEU model and, thus, estimate a subjective second-order belief for each subject.^4^ Besides its analytical features discussed in Sect. 3, our choice of the smooth ambiguity model is partly motivated by the findings of Attanasi et al. (2014) and Cubitt et al. (2017). See the latter paper’s Sect. 2 for discussion of literature in which these models are tested.

Dimmock et al. (2015) also assesses ambiguity sensitivity by estimating a form of α-MEU model, in which the set of probabilities is restricted to be a parametrised neighbourhood of some reference probability whose parameter captures the subject’s degree of uncertainty about probabilities. The estimation of the latter parameter marks a similarity to our approach to the extent that both estimate some aspect of beliefs about first-order uncertainty. However, there are several differences. Their approach is to estimate parameters of a special case of the α-MEU model, whereas we use the smooth ambiguity model. Moreover, for much of their analysis, they suppress differences across subjects in beliefs about uncertainty, taking these to be given by a shared neighbourhood of an assumed reference first-order probability. In contrast, we deliberately induce heterogeneity of second-order beliefs and estimate the latter separately for each subject, with only the support of such beliefs imposed. Finally, they elicit preferences using matching probabilities whereas we use certainty equivalents.^5^ The latter approach is natural given our objective, as a subject’s ambiguity premium is defined as the difference between two certainty equivalents.

We end this section by considering previous studies that have reported measures more directly akin to the ambiguity premia that we measure. Table 3 in Camerer and Weber (1992) lists a range of classic studies going back to Becker and Brownson (1964) which report similar measures by implementing the Ellsberg 2-colour/2-urn example. Essentially, they report the difference between the certainty equivalents of the bet on the draw from the “known” urn and the draw from the “unknown” urn, expressed as a percentage of the expected value of the objective lottery corresponding to the draw from the known urn. More recently, Trautmann and van de Kuilen (2016) have updated the table reported in Camerer and Weber (1992). Although they observe considerable heterogeneity in the ambiguity (and risk) premium reported across studies, the typical ambiguity premium is positive for all studies considered with a mean of roughly 14%, as compared to a mean risk premium of about 17%. Evidently, there is an affinity between the older literature on ambiguity premium and the current paper, but the older literature typically does not assume or estimate any particular model and, as a consequence, typically no beliefs are estimated. Failing to control for different beliefs across individuals would not be appropriate in our context because we wish to capture heterogeneity of second-order probabilities, and allow for it in assessing the strength of ambiguity sensitivity. Such allowance is required by the more refined conception of ambiguity premium that we use. While Abdellaoui et al. (2011) do estimate subjective beliefs, they concentrate on reporting ambiguity attitude in terms of the source functions they fit their data to. While a source function is undoubtedly a richer description of attitudes toward ambiguity, it is somewhat model specific; unlike the ambiguity premium, it is not a measure that is readily compared across models, nor with risk premium.

## The smooth ambiguity model and our experimental design

### Preference representation

Formally and as is standard, we represent the agent’s possible choices as acts, i.e. as maps from contingent states *s* ∈ *S* to consequences. In the smooth ambiguity model, an act *f* is evaluated by:

$$ E_{\mu } \phi \left( {E_{p \in P} \left( {u\left( f \right)} \right)} \right), $$

where *u* is a utility function measuring attitudes to objective risk, $\phi$ is an increasing function mapping utilities to reals, and $\mu$ is a second-order subjective probability over first order probability distributions on *S.* A first order probability measure is denoted by $p \in P$. The operators $E_{\mu }$ and $E_{p}$ take expectations with respect to the measures $\mu$ and $p$, respectively. Thus, $\mu$ represents the DM’s subjective uncertainty about the different first order probability distributions she deems possible (i.e. those in $P$) and, in this sense, is a *second*-*order belief*. In the model, this belief is captured by a (second-order, subjective) probability.^6^ Attitudes towards ambiguity are characterized by the shape of $\phi$. In particular, a concave (convex) $\phi$ characterizes ambiguity aversion (seeking), equivalently, an aversion (attraction) to mean preserving spreads in the distribution over expected utilities that is induced jointly by $\mu$ and $u$. When $\phi$ is linear or $\mu$ is degenerate, the smooth model collapses to a subjective expected utility model.

### Design core I: risky and ambiguous acts

In our experiment, subjects evaluated various acts, some of which were risky and others ambiguous. At the beginning of each session, subjects were shown some (opaque) white bags and told that coloured balls would be drawn publicly from these bags at the end of the session. All acts would be resolved by one of these draws and would yield €16 if a ball of a particular colour (which would vary by act) was drawn; and zero otherwise. Acts were presented to subjects as clauses of text of the form “if ball is ….. €16.00; if ball is …. €0.00”, with the two placeholders filled by colours and with a clear indication provided of which bag was referred to. Here, we report only tasks involving acts to be resolved from bags containing balls of two colours.^7^

One of these white bags (denoted ‘risky bag 1’ here) was publicly filled at the start of the experiment with 7 orange and 3 blue balls. Then, each subject’s certainty equivalent was elicited for an act yielding €16 if an orange ball was drawn from this bag. This procedure was repeated in an identical way with another white bag (hereafter ‘risky bag 2’), except that the contents of this bag were 3 orange and 7 blue balls. As the contents of risky bags 1 and 2 were known to subjects, the acts for which certainty equivalents had been elicited up to this point were risky, but not ambiguous. In contrast, the third and fourth acts were ambiguous, as we explain next.^8^

For these acts, subjects were shown a black bag (hereafter ‘the black bag’) that, as subjects were told, contained 12 balls, all numbered either 1 or 2. Subjects were not told the proportions of the two types of ball in the black bag. Before the elicitation of any further certainty equivalents, a ball was publicly drawn at random from this bag and, without its number being shown to either subjects or the experimenter, placed in a further white bag which we will call here ‘the ambiguous bag’.^9^ Subjects were told that, at the end of the experiment, the number on the ball in the ambiguous bag would be revealed and that bag would then be filled with the contents of whichever risky bag matched that number. Then, a final ball (which obviously would be either orange or blue) would be drawn from the ambiguous bag. Since tasks involving bets on orange and bets on blue were equally likely to be paid (see below), it was transparent that ‘rigging’ the ambiguous bag would not have been in the experimenters’ interests, even if it had been possible. More generally, it is a deliberate feature of our design that the uncertainties associated with the ambiguous bag were resolved by well-defined, physical events, exogenous to subjects’ behavior.

The simple and concrete nature of the procedure that determined the final contents of the ambiguous bag was designed to make clear that those contents would be either the contents of risky bag 1 or those of risky bag 2, thereby controlling the support (but only the support) of subjects’ second-order beliefs. Importantly, at the point when the relevant certainty equivalents were elicited, subjects did not know which risky bag would provide the contents of the ambiguous bag (and, as subjects could see, nor did the experimenters).^10^ Thus, acts resolved by draws from this bag were ambiguous, as the probabilities of different colours being drawn from it were unknown.

Subjects were not told the contents of the black bag, a draw from which would determine the contents of the ambiguous bag, but they did have some information about this. This information was allowed to vary across subjects and manipulated according to procedures which we describe in Sect. 3.4. First, we apply the smooth ambiguity model to the setup just described.

### Using the smooth ambiguity model to calculate ambiguity premia

As a convenient notation let, for *i* **=** 1, 2, risky bag *i* contain a proportion *p*_*i*_ of orange balls and a proportion 1 − *p*_*i*_ of blue balls. As explained in the previous sub-section, which of the two risky bags would be used to fill the ambiguous bag would depend on the number on a ball drawn from the black bag whose composition was unknown to subjects, except that it contained balls numbered 1 or 2.

Consider, to begin with, the position of a subject not knowing the number on the ball drawn from the black bag. Assuming smooth ambiguity preferences, the certainty equivalent of the bet that an orange ball is drawn from the ambiguous bag, ${\text{CE}}_{\text{Amb}} ({\text{orange}})$, satisfies the following equation:

$$ \phi \left( {u\left( {{\text{CE}}_{\text{Amb}} \left( {\text{orange}} \right)} \right)} \right) = \phi \left( {p_{1} u\left( {c^{*} } \right) + \left( {1 - p_{1} } \right)u\left( c \right)} \right)\mu + \phi \left( {p_{2} u\left( {c^{*} } \right) + \left( {1 - p_{2} } \right)u\left( c \right)} \right)\left( {1 - \mu } \right), $$

where the tuple $\left( {\mu ,\phi ,u} \right)$ characterizes the parameters fixing the subject’s preferences, and $c^{*}$ and $c$ are the payoffs in case the bet succeeds or fails. (Similarly, for ${\text{CE}}_{\text{Amb}} ({\text{blue}})$, with $c^{*}$ and $c$ transposed.) For any subject, beliefs are fully characterized under the smooth ambiguity model by a single parameter $\mu$, interpretable in the current context as the subject’s belief about the proportion of balls in the black bag that are numbered 1, a proportion that the subjects do not know.^11^ Now, consider the (hypothetical) position of a subject who *did* know which ball had been drawn from the black bag, and hence which of the two risky bags would be used to fill the ambiguous bag. (Of course, this hypothetical position is the same as the actual one of a subject facing one of risky bags 1 or 2.) In this case, the (second-order) belief *μ* is degenerate on *p*_*i*_, the actual proportion of orange balls in the relevant risky bag $i$, and the certainty equivalent of the bet that an orange ball is drawn, ${\text{CE}}_{i} ({\text{orange}})$, satisfies the following equation:

$$ u\left( {{\text{CE}}_{i} \left( {\text{orange}} \right)} \right) = p_{i} u\left( {c^{*} } \right) + \left( {1 - p_{i} } \right)u\left( c \right), $$

(and, again, similarly for blue).

We assume that utility is given by $u(x) = x^{1 - \rho } /(1 {-}\rho )$, and $\phi$ by $\phi (x) = {-}e^{ - \varphi x} / \varphi$. With these specifications, the parameters $\rho$ and $\varphi$ represent the coefficient of relative risk aversion (CRRA) and the coefficient of absolute ambiguity aversion (CAAA), respectively and, jointly with $\mu$, completely parametrize a subject’s preferences.

For a subject who conforms exactly to the current assumptions and whose certainty equivalents are observable precisely, either of the certainty equivalents ${\text{CE}}_{i} \left( {\text{orange}} \right)$ or ${\text{CE}}_{i} \left( {\text{blue}} \right)$ would yield the parameter $\rho$ and the two certainty equivalents ${\text{CE}}_{\text{Amb}} \left( {\text{orange}} \right)$ and ${\text{CE}}_{\text{Amb}} \left( {\text{blue}} \right)$ would then provide sufficient information to infer the remaining elements ($\varphi$ and $\mu$) of the parameter triple for each subject. Apart from the functional form of the preference, the key identifying assumption is that the probabilities $p_{i}$ that the subject considers possible are those that the instructions tell them are possible (and which actually are those possible). Beyond this, there is no restriction on beliefs: $\mu$ is obtained from the data, not imposed.

In practice, we elicited certainty equivalents for acts using choice-lists presented to subjects on computer screens. For each act, subjects were asked to make 21 choices between the act and each of an ascending range of certain amounts grouped together in a list, ranging from 0€ to 16 €, and increasing in in equal steps of 0.80€. For a given act, the midpoint of the last choice in which the subject chose the act and the first choice in which the subject chose the certain amount was taken as the certainty equivalent, when required. Since in our experimental design $p_{2} = 1 - p_{1}$ we presented subjects with choice lists from which certainty equivalents could be approximated in this way for bets on an orange ball being drawn from each of risky bags 1 and 2, and then for bets on each of orange and blue being finally drawn from the ambiguous bag. For each subject individually, the resulting data was used in two ways. First, we estimated the parameter tuple $\left( {\mu ,\varphi ,\rho } \right)$ from the choices. In particular, for each subject, we used maximum likelihood to maximize the probability of observing the 84 choices in the relevant choice-lists by minimizing the differences between the valuation of the act and the valuation of the certain amount divided by a Fechnerian error term (e.g., Hey and Orme 1994). Second, we used the estimated parameters and the certainty equivalents for the ambiguous acts to obtain ambiguity premia for those acts, as we now explain.

Recall that the ambiguity premium is a hypothetical ambiguity-neutral agent’s certainty equivalent for the act minus that of the real agent, with the hypothetical agent assumed to share the actual agent’s subjective information and attitude to objective risk. Thus, under the smooth ambiguity model, a subject’s ambiguity premium for the bet that an orange ball is drawn from the ambiguous bag is given by $X - Y$, where

$$ \begin{aligned} X & \equiv u^{ - 1} \left( {\left( {p_{1} u\left( {c^{*} } \right) + \left( {1 - p_{1} } \right)u\left( c \right)} \right)\mu + \left( {p_{2} u\left( {c^{*} } \right) + \left( {1 - p_{2} } \right)u\left( c \right)} \right)\left( {1 - \mu } \right)} \right),\quad   {\text{and}} \\ Y & \equiv {\text{CE}}_{\text{Amb}} \left( {\text{orange}} \right). \\ \end{aligned} $$

The term $Y$ is directly observed, as it is the subject’s actual certainty equivalent for the relevant ambiguous act. In contrast, the term $X$ is not observed as it is the certainty equivalent of the hypothetical agent. Instead, $X$ is computed from the estimates of $\rho$ and $\mu$ using the fact that an ambiguity-neutral agent may be represented by setting $\phi \left( x \right) = x$. (The values of c* and *c* and, under our identifying assumption, *p*_1_ and *p*_2_ are known to the experimenter.) As the hypothetical ambiguity-neutral agent shares the risk attitude and beliefs of the real agent, the parameters $\rho$ and $\mu$ are reflected in the constructed certainty equivalent *X*. To this extent, the ambiguity premium *X* − *Y* controls for risk attitude and beliefs.^12^ Three features of this approach are worth stressing at this point. First, the act for which $X - Y$ gives the subject’s ambiguity premium is a *first*-*order* act, i.e. a bet on a draw from the ambiguous bag. Second, it is our use of the smooth ambiguity model that allows us to estimate a *second*-*order* belief parameter ($\mu$) (with a natural interpretation in our design) and to use it in constructing the hypothetical agent’s certainty equivalent $\left( X \right).$ Third, the hypothetical agent shares not just $\mu$, but also attitude to risk with the real agent. Hence, in general, $X$ is not simply the expected value of the act, conditional on $\mu$, $p_{1}$, and $p_{2}$.

### Design core II: treatments and information heterogeneity

To induce heterogeneity in second-order beliefs among subjects, we used both a treatment variation and a device designed to induce heterogeneity within each treatment.

The treatment variation manipulated the actual composition of the black bag. Specifically, there were three treatments, each featuring a different proportion of balls in the black bag numbered 1. This proportion was either 0.25, 0.5 or 0.75, with the variation administered between sessions. Below, we use these values as the names of the treatments.

Different subjects within the same session could also have different information about the black bag because, in each treatment, subjects were allowed short private peeks into it, thereby obtaining imperfect, private signals of its contents. These peeks were separate for each subject, with the bag being shaken by the experimenter between peeks to ensure that each subject received a different visual signal. As well as inducing heterogeneity, this device served to emphasise the imperfect nature of the subject’s information about the relative likelihood of the different possible compositions of the device that would resolve the ambiguous acts.

The existing literature is populated mostly by designs where subjects are told nothing about the resolution of the ambiguous acts beyond the possible first-order probability distributions (i.e., the support of the second-order distribution, as e.g., in Gneezy et al. 2015) and designs where subjects are told everything [i.e., the entire second-order distribution as in Halevy (2007)]. Here, we explore an information environment that is more realistic, something in between nothing and everything.^13^

Perhaps the closest that the previous literature comes to our focus on incomplete and potentially heterogeneous subjective information is the “bingo blower” design (Hey et al. 2010; Hey and Pace 2014; Carbone et al. 2017). This design provides subjects with incomplete information about objective probabilities via a visual impression of a blur of moving coloured balls, one of which will be drawn. It is natural to suppose that this gives each subject some idea of the probability that the ball drawn has a given colour and perhaps that there may be variation across subjects in this idea, for example because of different angles or timing of view. However, as noted above, these papers are concerned with comparison of theories, rather than calculation of ambiguity premia. The design reported by Oechssler and Roomets (2015) also uses a mechanical device, in their case one constructed by subjects, to generate an uncertain composition for an Ellsberg urn. Their main concern is to classify subjects by whether they are ambiguity-averse or not, rather than to assess the strength of ambiguity sensitivity.

### Other procedures

As described above, certainty equivalents for acts were elicited with choice-lists presented to subjects on the computer screen. (Appendices B and C contain experimental instructions and an example choice-list, respectively.) For each subject, one row of one choice-list was randomly selected at the end of the experiment and paid out for real. That is, if the a subject chose the certain amount in the randomly selected choice, (s)he would receive the certain amount. Otherwise, the subject received the outcome of the act depending on a ball drawn from the relevant bag, with all these draws made at the end of the experiment.^14^

The experiment was conducted at Tilburg University with a total of 88 undergraduate students, in sessions of approximately ten students. Subjects were first presented with instructions. Then, after a practice, certainty equivalents of the various acts were elicited and outcomes determined, as explained above. As well as receiving the outcome of the choice selected to be for real for them, subjects received a show-up fee of €5. All payments were made immediately in private and in cash. On average, subjects earned €13.96. The experiment was programmed in z-tree (Fischbacher 2007).

## Experimental results

Four subjects were excluded from the analysis because they switched multiple times between the act and the certain option. For each of the remaining subjects, we estimated the parameter tuple $\left( {\mu ,\varphi ,\rho } \right)$ and then computed ambiguity premia in the way described above. Here, we report the estimated parameters first, then the ambiguity premia.

### Risk attitudes

The median estimated coefficient of relative risk aversion is equal to 0.066, revealing significant risk aversion (*p* value = 0.047), and in line with previous findings in the literature (e.g., Abdellaoui et al. 2007; Ahn et al. 2014). When using the estimated CRRA coefficient to classify subjects according to their attitude towards risk, we find that 51.2% of subjects are risk averse, 27.4% risk seeking, and 21.4% risk neutral.^15^

### Ambiguity attitudes and beliefs

The median estimate of the CAAA coefficient is 0.079, which is significantly larger than zero, i.e., the median subject is significantly ambiguity-averse (*p* value = 0.016). Also, Mann–Whitney tests reveal that ambiguity attitudes, as captured by this parameter, do not differ between treatments at conventional levels of significance (treatment 0.75 vs. 0.5: *p* value = 0.730; treatment 0.75 vs. 0.25: *p* value = 0.092: treatment 0.5 vs. 0.25: *p* value = 0.217). Classifying subjects on the basis of the CAAA coefficient yields the finding that 53.57% of subjects are ambiguity-averse, 27% ambiguity-seeking, and 21.43% ambiguity-neutral.^16^ These numbers are broadly comparable with other similar experiments (e.g. Cohen et al. 2011; Dimmock et al. 2015; Oechssler and Roomets 2015).

**Fig. 1:**
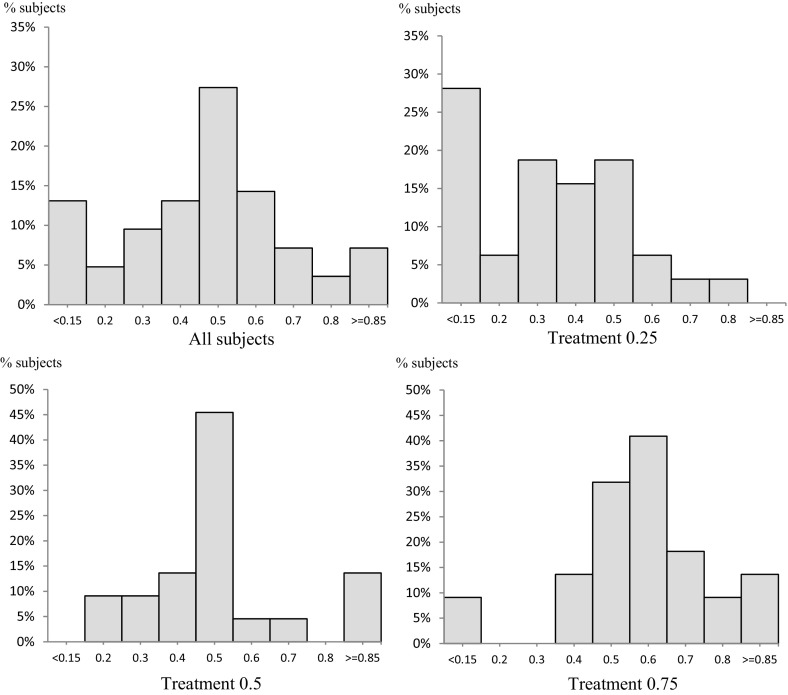
Revealed belief

We turn now to the distribution across subjects of the revealed belief $\mu$. We find very considerable heterogeneity of the revealed $\mu$ both across and within treatments. This can be seen from the histograms in Fig. 1, showing the distribution of revealed $\mu$ for each treatment, and for all subjects together. In the figure, the height of a single bar of a histogram shows the proportion of subjects whose revealed belief lies in the interval defining the base of the bar. Thus, for example, taking all treatments together, 27.4% of subjects revealed a value of $\mu$ in the central interval 0.45 ≤ $\mu$ ≤ 0.55; and many of the departures from centrality are quite marked, with 28.6% of subjects in the four outer columns.

The within treatment heterogeneity of beliefs is clear from the panels for each treatment, with widely dispersed values of $\mu$ present in every case. This indicates that, in each treatment, subjects could not easily infer the true proportion of balls in the black bag numbered 1 and therefore is indirect evidence that our design did create ambiguity, despite its structured set-up.

Yet, notwithstanding the ambiguity, the peeks did convey some information to subjects, as is evident from the systematic way in which beliefs differ across the three treatments in the expected directions. Mann–Whitney tests reveal that subjective beliefs $(\mu )$ in the treatment with objective probability 0.25 were smaller than those in the 0.5 treatment (*p* value = 0.004), which were in turn significantly smaller than revealed beliefs in the treatment with objective probability 0.75 (*p* value = 0.026). Moreover, the panel for the 0.5 treatment shows a broadly symmetric distribution around the true proportion of balls in the black bag numbered 1, but the panels for treatments 0.25 and 0.75 show positive and negative skew, respectively.

Putting these points together, the peeks seem to have served their purpose as imperfect, private signals, conveying information, but ambiguous information. Note that it is our use of a model in which second-order belief is separately parametrized that allows us to report this check.

### Ambiguity premia

**Fig. 2:**
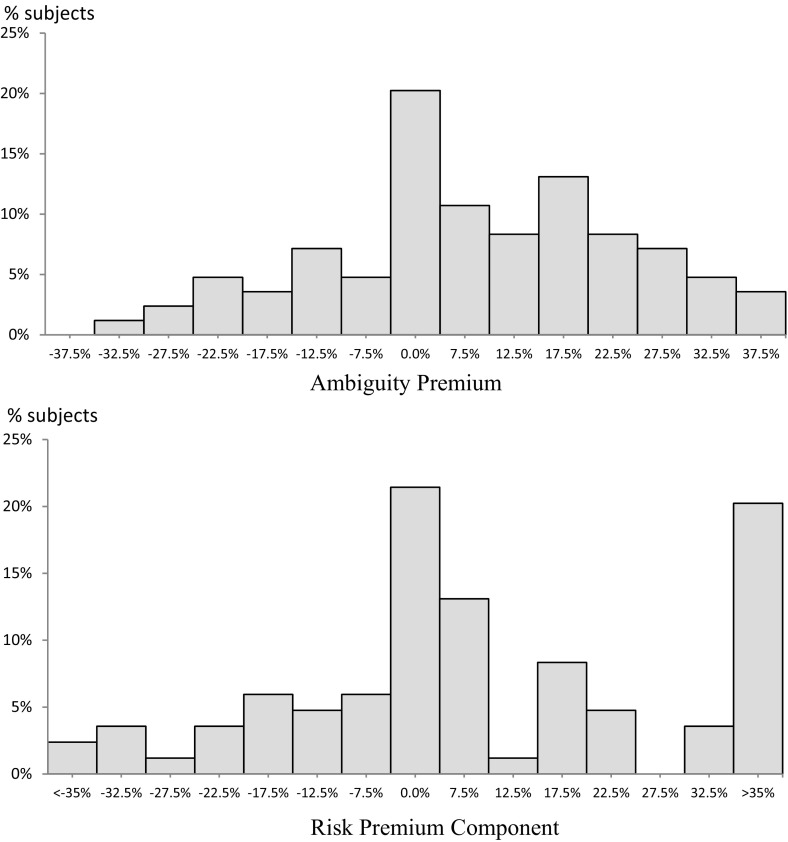
Ambiguity premium (top) and risk premium component (bottom)

Our main reason for estimating subjects’ second-order beliefs was to calculate their ambiguity premia, to which we now turn. For each subject, for each of the two^17^ ambiguous acts, we calculated the ambiguity premium for that act using the subjective belief ($\mu$) and attitudes towards risk ($\rho$) estimated for the subject, as explained at the end of Sect. 3.3. In order to compare our results to findings in the literature, we present our results in the form of a normalized ambiguity premium, expressing the premium itself (for a given subject and a given ambiguous act) as a percentage of the expected value of the act, where the expectation is calculated using the estimate of $\mu$ applicable to the subject. The average of the two normalized ambiguity premia, so computed for a given subject, is what we take as the ambiguity premium associated with the subject. The top histogram of Fig. 2 shows the distribution of the ambiguity premium across all subjects and Table 1 gives summary statistics by treatment.

**Table 1:** Ambiguity premium across treatments

	Treatment			All (*n* = 84), %
	0.25 (*n* = 32), %	0.5 (*n* = 22), %	0.75 (*n* = 30), %	
Mean	8.60*	2.36	9.07*	7.13*
Median	11.50*	4.50	9.50*	7.00*
Standard deviation	16.15	18.06	15.89	16.62

*Significantly larger than zero at the 5% significance level using a *t* test (for means) or Wilcoxon signed-rank test (for medians)

As can be seen in Table 1, the resulting mean and median ambiguity premium for all subjects taken together are approximately 7.1 and 7.0%, respectively, both significantly different from zero. Yet, the top panel of Fig. 2 shows a large amount of heterogeneity around these central tendencies. Though a majority of those departing from the central category do so in an upward direction, there are large departures in both directions. On average, the strength of attitudes towards ambiguity manifested in the ambiguity premium is somewhat lower than is typically observed in existing studies (see Camerer and Weber 1992 and Trautmann and van de Kuilen 2016). This might be explained by the fact that many previous studies do not estimate a model and, more importantly, do not control for the role of (heterogeneous) beliefs, as we do. This might have introduced an upward bias to their estimated ambiguity premium if beliefs concerning the ambiguous event were pessimistic. From this perspective, it is notable that we observe significant and quantitatively plausible aversion to ambiguity even after controlling for subjective beliefs at the individual level.

Though there are some differences between treatments in raw central tendencies of the ambiguity premium, these do not reach conventional levels of statistical significance. Appendix A gives histograms for ambiguity premium, broken down by treatment, providing a visual indication that any differences between treatments in terms of ambiguity premia are quite subtle. This is encouraging, as the purpose of the treatments was to induce heterogeneity of beliefs, not necessarily of ambiguity premia. This is consistent with the premia reflecting *attitude* to ambiguity, rather than beliefs about uncertainty, as the formula we use for ambiguity premium controls for the subjective belief $\mu$.

**Fig. 3:**
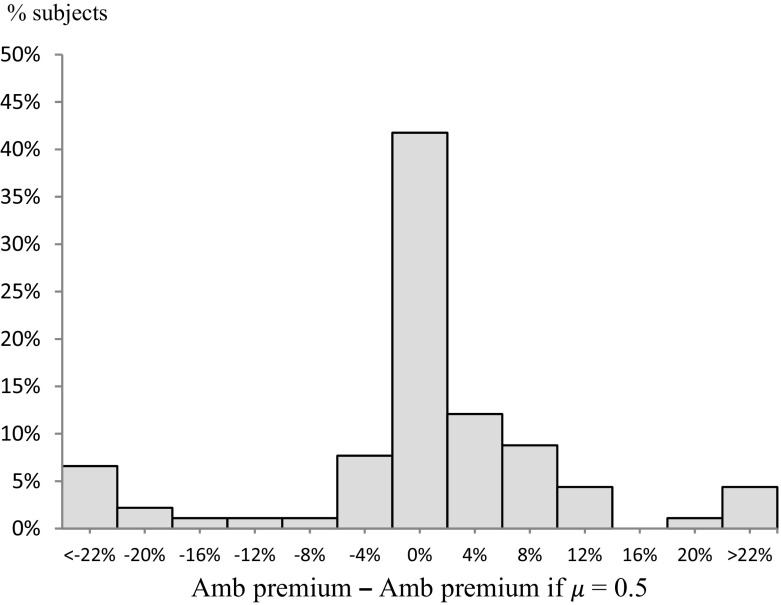
The impact of estimating $\mu$

It is therefore natural to ask what difference it would have made if, instead of estimating and controlling for $\mu$ in this way, we had simply assumed it to be uniform. In our context, this would have amounted to assuming $\mu$ = 0.5 for each subject. Figure 3 gives the distribution across subjects of the *difference* between ambiguity premium as we calculate it and the premium that would have resulted had we assumed $\mu$ = 0.5. The central column of Fig. 3 reflects the fact that, for c. 45% of subjects, estimating $\mu$ rather than assuming it makes a difference of less than ± 2% points to the ambiguity premium. But, for the majority the difference is larger. In fact, for 31 out of 84 subjects, the ambiguity premium with $\mu$ estimated differed from that with $\mu$ assumed to be 0.5 by at least 5% points. This reinforces the value of estimating $\mu$, especially as we are concerned with the distribution of ambiguity sensitivity, not just its central tendency.

Finally, it is interesting to compare the extent to which a subject’s evaluation of an ambiguous act is affected by ambiguity attitude with the extent to which that evaluation is affected by risk attitude. Consider the overall uncertainty premium for an act, defined as the difference between the observed certainty equivalent of act and its expected value (calculated using estimated beliefs). If we subtract the ambiguity premium from the uncertainty premium, the residual gives the extent to which the evaluation of the act is notionally affected by the (revealed) risk and risk attitude. Normalizing this residual by the expected value we obtain the normalized risk premium component. The distribution of this component is shown in the lower panel of Fig. 2. The mean, median and standard deviation of this normalized risk premium component were found to be 11.89, 5.23 and 31.80%, respectively, quite similar to the corresponding numbers for ambiguity premium. Indeed, a signed-rank test reveals that the normalized risk premium component is not significantly different from the (normalized) ambiguity premium (*p* value = 0.865). This suggests that, among the subjects of this experiment, the strength of ambiguity sensitivity is roughly the same as the strength of risk sensitivity, in the sense that on average they contribute in roughly equal measure to the uncertainty premium.

However, that is not to suggest that a subject’s risk and ambiguity premium components are positively correlated. In fact, we found a small but significant negative correlation. This is broadly in line with some findings in the literature (e.g. Sutter et al. 2013). However, Camerer and Weber (1992) point out most early studies find ambiguity and risk attitudes to be largely independent; and some more recent studies, e.g., Abdellaoui et al. (2011), and Bossaerts et al. (2009) find a significant positive correlation.

## Concluding remarks

This paper reports an experimental investigation into the strength of ambiguity sensitivity, based on using choices to elicit individuals’ certainty equivalents for uncertain acts to be resolved by a variation on the famous Ellsberg 2-colour, 2-urn example; and then using the resulting data to calculate each subject’s ambiguity premium for each uncertain act. The choices were fitted to the smooth ambiguity model yielding estimates, subject-by-subject, of the three parameters of that model, one governing each of: the (second-order) belief, ambiguity attitude, and risk attitude. The parameter estimates were applied to deconstruct each subject’s certainty equivalent for a given uncertain act into an ambiguity premium component and a risk premium component. The presence of a second-order belief in the parametrisation offered by the smooth ambiguity model is crucial to this exercise.

In our setting, subjects are not faced with an Ellsberg urn and told nothing more about it than the possible compositions. Instead, by design, they have some, but heterogeneous and imprecise, information about the likelihoods of different compositions, making the setting more realistic in this respect. We find that the median ambiguity premium is 7%. Though this figure is lower than some of those in previous literature, it is economically significant and of the same order of magnitude as the median risk premium component (5.2%). Moreover, we find a wide variation in ambiguity premia between subjects, indicating a corresponding variation in ambiguity attitude, since our measure controls for the substantial heterogeneity of beliefs that we also find. The existence of variation in ambiguity attitude is economically significant in its own right, because it indicates scope for ambiguity-sharing as well as the risk-sharing that is typically taken to account for much of the activity of the modern financial economy. The heterogeneity of second-order beliefs that we find is also important as it reinforces our claim that subjects faced real ambiguity in our design.

Besides these findings, we also demonstrate the feasibility of using the framework of the smooth ambiguity model and data on decisions involving first-order acts to control for subjects’ second-order beliefs about the uncertainty facing them, when quantifying their sensitivity to that uncertainty; and we give a numerical indication of the value of doing so. For more than half of our subjects, controlling for beliefs alters our computation of their ambiguity premium by 2 or more percentage points, relative to the figure we would have obtained if we had followed previous studies in assuming uniform beliefs.

## Appendix B: Instructions

**Key to readers of paper**: The instructions given below are those used in the experimental sessions. The following translates the terminology used in the paper to that used in the instructions:

“Risky bag 1” of the paper is “Bag 1” of the experimental instructions.

“Risky bag 2” of the paper is “Bag 2” of the experimental instructions.

The “ambiguous bag” of the paper is “Bag 6” of the experimental instructions.

The “black bag” of the paper is the “black bag” of the experimental instructions.

The experiment also included tasks not relevant to the present paper, involving “bags 3, 4, 5 and 7” of the experimental instructions. Passages of the instructions that relate only to these tasks are omitted below, except for indicators of where these passages were. The non-bold text below was distributed to subjects on paper and read aloud by the experimenter. The bold text comprises instructions to the experimenter and was not given to subjects.

### Instructions

**As subjects arrive:**

**Check them against list of participants. When all arrived or start-time reached, close door read the following preliminary announcement:**

Welcome to the CentER Experimental Laboratory. Thank you for coming to this experiment, which is part of a joint research project by staff at the Universities of Nottingham, Oxford and Tilburg.^18^ In a moment, I will tell you more about it, but first I need to set some ground-rules.

Please switch off your mobile phones. Do not attempt to use the computers for any purpose other than the experiment.

If you encounter a difficulty during the experiment, for example, if there is anything you do not understand, you’re welcome to ask a question. But, rather than calling out, please raise your hand and one of us will come to attend to you. Please do not attempt to solve your difficulty by looking at the computer screens of other participants.

We expect that the experiment will take about 1 h, but it may take a little more or less than that. It is essential that participants stay till the end. If you are not able to do so, please say so now.

At the end of the experiment, we will pay you a fixed sum of 5 EUR for attending, provided you complete all the tasks and a short questionnaire. In addition, the experimental tasks will give you the chance to receive a further payment. How large that payment is will depend partly on your own decisions and partly on chance. No subject’s decisions will affect the payment received by any subject other than themselves.

Here we have a deck of cards. The numbers on the cards correspond to seats in the laboratory. Please take a card and be seated at the computer corresponding to the number on the card.

**When everybody is seated, read the following aloud:**

I am now going to read you some instructions for the tasks. There is a copy of those instructions on your desk that you may consult at any time.

### Opening description

During the experiment, you will make several choices, involving amounts of money and chances. At the end of the experiment, your computer will select one of your choices at random for payment. You will be paid on the basis of what you chose in that task, after any chances have been resolved.

Note that, although only one choice will actually determine the payment we make to you, it could be *any* of the choices. Thus, every choice you make could affect your payment. Whatever payment you are due will be paid to you in cash at the end of the experiment in private.

Each task is a choice between two options. Some of the options yield amounts of money that depends on the colour of a ball drawn at random from a white bag like this one, containing balls like these. **[Show bag and balls.]**

We will use seven different bags, labelled 1, 2, 3, 4, 5, 6, and 7. Each white bag will contain a set of coloured balls that may differ in their composition from bag to bag. At the end of the experiment, we will actually draw one ball from each of the white bags.

### Description of a choice list

The choices will be grouped together in lists that you will see on your computer screen. Here is a screenshot of an example choice list:

**Written version of instructions has a screenshot of an example choice list involving a risky bag X with 5 orange balls, 5 blue balls, and 5 green balls.**

This is not one of the actual choice lists from the experiment, but just an example to show you their format.

The list consists of several choice tasks. Each choice is described on one line of the table and is between two options: Option A and Option B.

Option A yields an amount of money that depends on the colour of a ball drawn from bag X whereas Option B yields a particular amount of money for sure.

For example, in Choice 11, Option A yields 10 EUR if the ball drawn from bag X is orange and zero if it is blue or green; whereas Option B yields 5 EUR for sure. So, if Choice 11 was the task selected to determine your payment, you would be paid 5 EUR, if you chose Option B; and either 10 EUR or zero, depending on the colour of the ball drawn from bag X at the end of the experiment, if you chose Option A.

There are several points that you should note about the structure of a choice list.

At the top of the choice list, you can see information about the contents of the bag. In this example, this information tells you that bag X contains 5 orange balls, 5 blue balls, and 5 green balls.

Now look at the options. Note that Option A is the same for each choice in the list. In this example, it always yields 10 EUR if the ball drawn from the bag is orange and zero if it is blue or green.

In contrast, Option B yields a particular amount of money for sure. Note that this amount of money is different in each choice in the list. In the first choice, it is zero. But, the amount rises in even steps as you move down the choice list. In the final choice in the list, the sure amount of money given by Option B is equal to the maximum that can be won under Option A.

We imagine that most people will choose Option A for some of the choices in the list and Option B for others. In particular, for this example, we imagine that most people would choose Option A in Choice 1 since, in that choice, Option A gives a chance of a sum higher than zero, whereas Option B gives zero for sure. We also imagine that, as the amount of money offered by Option B rises, most people would switch to Option B at some point. In this example, by Choice 21, Option B gives 10 EUR for sure, whereas Option A only gives a chance of 10 EUR. So, we imagine most people would choose Option B in Choice 21. Note, however, that it is entirely up to you when to switch from Option A to Option B and, indeed, what to do in each of the choices. Just remember that each one of the choices could prove to be the one that determines the payment you receive; at the end of the experiment, the computer will randomly select one choice list and one of your choices from that list for payment. You will be paid on the basis of what you chose in that task, after any chances have been resolved.

The way that you complete a choice list is by using your mouse to select either Option A or Option B, in the right-hand column for each choice on the list. You cannot continue to the next list until you have selected an option for every choice. Once you have done this and are satisfied with your choices, click on the Continue button to proceed. Note that, once you have clicked on Continue, you will not be able to change your choices.

You will complete several choice lists during the experiment. They will differ from each other, but will all have the same basic structure. In particular, although the options will vary from list to list, the outcome of Option A will always depend on a draw from a bag, whereas Option B will always be a sure amount of money. Within each list, Option A will be the same on each row, whereas Option B will rise from zero in even steps to the maximum that can be won under Option A.

There are a number of points that will vary from one choice list to another. In particular, the composition of the bag and the information that you have about the composition will vary from one choice list to another. Also, the winning-colour under Option A will vary. You should pay attention to all these points, for each choice list.

Are there any questions at this moment?

**Allow a moment for questions. Then proceed to …..**

**Construction of BAG 1**

All right, here is a white bag, which we will call Bag 1. I am going to place 7 orange balls and 3 blue balls in BAG 1. **[Show balls and put in the bag. On whiteboard, write “Bag 1** **=** **7 orange and 3 blue balls”.]** When the time comes to draw a ball from Bag 1, I will ask one of you to randomly draw a ball from the bag. Your computer will now display some choice lists involving Bag 1. Please complete them at your own pace.

**Construction of BAG 2**

All right, here is another white bag, which we will call Bag 2. I am going to place 3 orange balls and 7 blue balls in BAG 2. **[Show balls and put in the bag. On whiteboard, write “Bag 2** **=** **3 orange and 7 blue balls”.]** When the time comes to draw a ball from Bag 2, I will ask one of you to randomly draw a ball from the bag. Your computer will now display some choice lists involving Bag 2. Please complete them at your own pace.

[*Note to readers of the paper*: For brevity, we omit the portion of instructions relating to tasks not reported in this paper. These instructions used three more risky bags, numbered 3, 4 and 5 at this point.]

**Construction of BAG 6**

Here is another white bag, which we will call Bag 6. You will not know for sure how many balls of each colour will be put in Bag 6, until the time comes to draw a ball from it. At that time, Bag 6 will be filled with the contents of either Bag 1 or Bag 2.

Which bag it is, will be determined as follows.

You can see that I have a black bag here. The black bag is filled with numbered balls. Each ball in the black bag has a number on it, either 1 or 2. I am not going to tell you how many balls there are of each number, but in a second, I will come to your table and allow each of you to take a 5-second peak in the black bag. You can look in the bag, but are not allowed to touch it or try to empty it out. Between each peak, I will shake the bag.

After you have all taken your peak in the black bag, I will draw one of the numbered balls from it, at random and without looking, and will put that ball into Bag 6. When the time comes to draw a coloured ball from Bag 6, I will first remove the numbered one from it and one of you will read aloud the number on that ball. If the number is 1, I will fill Bag 6 with the contents of Bag 1; and, if the number is 2, I will fill Bag 6 with the contents of Bag 2. One of you will then draw a coloured ball from Bag 6. Thus, when the coloured ball is drawn from it, Bag 6 will have the contents of either Bag 1 or Bag 2. However, none of us will know in advance which of those contents it will be.

**[On whiteboard, write: “Bag 6** **=** **EITHER Bag 1 or Bag 2”.]**

I will now come to your table and allow each of you to take a 5-second peak in the black bag.

**[Allow for peaks]**

**[Draw ball and put into bag 6]**

Your computer will now display some choice lists involving Bag 6. Please complete them at your own pace.

[*Note to readers of the paper*: For brevity, we omit the portion of instructions relating to tasks not reported in this paper. These instructions used another ambiguous bag, numbered 7, at this point.]

**Drawing of balls**

Thank you for completing the choice tasks. I will now ask one of you to draw balls from bags 1 till 5, and type the result in the computer… first bag 1.

**Drawing balls from bags 1 to 5. Type results in on computer.**

Now I will reveal the balls in bags 6 and 7, fill each of them with the contents of the bag corresponding to the number of the ball, and ask one of you to draw a ball from each of the bags.

**Reveal balls, fill bag 6–7, draw balls and type in on computer.**

Your computer will now display the results of the draws, the task that was elected for payment and your earning. If you have read them, you can click on continue.

**Paying out**

**[Computer reveals, for each subject, the choice that is real for them; what they chose and the outcome. Computer adds show-up fee.]**

Okay, while I make the receipts, we would like you to fill in a short questionnaire.

**[When they have completed the questionnaire, subjects are asked to remain seated until their seat number is announced. Then, they are asked to stand up and walk to the pay-out table, bringing their seat number with them.]**

## Appendix D: An alternative method

An alternative empirical strategy for identifying our preference parameters to the one that we have used in this paper would be to apply a matching probability method, akin to one explored in recent literature (e.g., Baillon and Bleichrodt 2015; Dimmock et al. 2015, 2016).

To illustrate what this alternative empirical strategy would have constituted, consider the two risky bags used in the experiment (i.e., risky bag 1 filled with 7 orange balls and 3 blue balls and risky bag 2 filled with 3 orange balls and 7 blue balls), and the ambiguous bag (i.e. a bag which may have either the contents of bag 1 or of bag 2). In addition, let us consider the ambiguous act that yields €16 if an orange ball is drawn from the ambiguous bag, and the ambiguous act that yields €16 if a blue ball is drawn from the ambiguous bag, as in our experiment. The *matching probability* of an ambiguous act for event *E* is defined as the probability *P* that makes the decision-maker indifferent between receiving €*x* with (known) probability *P* and receiving €*x* if event *E* occurs. In the current setting, the matching probability of each ambiguous event (i.e., of the events “an orange ball is drawn from the ambiguous bag” and “a blue ball is drawn from the ambiguous bag”) can be elicited in a straightforward manner by letting subjects make a series of choices between a risky act yielding either €16 or nothing with probability *q* and each of the two ambiguous acts, where *q* varies between 0 and 1 and observing the value of *q* that leads to indifference between the risky and the ambiguous act. Let us denote the matching probabilities of the two ambiguous acts by $P_{\text{Amb}} ({\text{orange}})$ and $P_{\text{Amb}} ({\text{blue}})$, respectively. Since expected utilities are linear in probabilities, the smooth ambiguity model guarantees that, with the normalization $u\left( {c^{*} } \right) \equiv 1$ and $u\left( {c_{*} } \right) \equiv 0$, these two matching probabilities imply $\phi \left( {\left( {P_{\text{Amb}} \left( {\text{orange}} \right)} \right)} \right) = \phi \left( {0.7} \right)\mu + \phi \left( {0.3} \right)\left( {1 - \mu } \right)$ and $\phi \left( {\left( {P_{\text{Amb}} \left( {\text{blue}} \right)} \right)} \right) = \phi \left( {0.3} \right)\mu + \phi \left( {0.7} \right)\left( {1 - \mu } \right)$, respectively. So, if one assumes a parametric form for $\phi$ like we do, one can calculate the value of parameters $\varphi ,\mu$, and separately, $\rho$ from the risky choices. Then, one would proceed to construct the predicted certainty equivalents *X* and *Y* defined in Sect. 3.3, using the values of $\varphi ,\mu$, and $\rho$ so obtained. Unlike in our method of Sect. 3.3 (where only the certainty equivalent of the *hypothetical* ambiguity-neutral agent (*X*) was constructed but *Y* was measured directly), both *X* and *Y* would be constructed in the alternative method just sketched.

## Footnotes

1 This objective contrasts with that of the large theoretical literature, surveyed in Etner et al. (2012) and Gilboa and Marinacci (2013), which provides models of ambiguity sensitive preferences. There is also a growing literature whose objective is to test models or compare their empirical performances. Some contributions in this vein provide estimates of parameters governing ambiguity sensitivity in particular models as a by-product and are therefore discussed below. Others, such as Attanasi et al. (2014), Baillon and Bleichrodt (2015), Binmore et al. (2012), Cubitt et al. (2017), Halevy (2007) and Hayashi and Wada (2010), adopt more qualitative approaches to testing.

2 See the classic Ellsbergian literature surveyed by Trautmann and van de Kuilen (2016). Oechssler and Roomets (2015, Table 1) reports the proportion of ambiguity-averse subjects from a range of studies between 1964 and 2015. See also Baillon et al. (2016b) for a recent study that includes such categorisation alongside more refined forms of ambiguity sensitivity. Chakravarty and Roy (2009) also focuses on detection of ambiguity aversion and seeking among their subjects, allowing that such attitudes may differ between the domains of gains and losses.

3 Though we concentrate on these approaches, there are others. For example, Cohen et al. (2011) and Charness et al. (2013) report measures of strength of ambiguity sensitivity that are based neither on model parameters nor on premia, but instead on directly-observed features of the data, such as the number of tasks in certain sets in which an unambiguous option is chosen, or detection of other patterns in responses. A different approach taken in some recent literature is to quantify ambiguity sensitivity using matching probabilities. We discuss this approach in the context of studies that use matching probabilities to estimate parameters of models.

4 Like Gneezy et al, Potamites and Zhang (2012) also measure ambiguity sensitivity through the parameter α of the α-MEU model. These papers also have in common that they discuss in more detail than we do the question of whether that parameter is correlated with attitude to objective risk. Stahl (2014) estimates parameters of a bespoke logistic choice model, which he terms the βγ-model. This model has a close affinity to the maxmin expected utility model of Gilboa and Schmeidler (1989), which in turn is a special case of the α-MEU model.

5 Matching probabilities are defined by indifferences between objective lotteries and particular ambiguous acts. Their uses are elaborated by Dimmock et al. (2016) and Baillon et al. (2017), which assess ambiguity sensitivity in large representative samples, and by Baillon et al. (2016a) which uses them to measure ambiguity attitudes for natural events. As these studies and Voorhoeve et al. (2016) illustrate, matching probabilities do not have to be used to estimate parameters of theoretical models, though that is the main use Dimmock et al. (2015) and Baillon et al. (2017) make of them. We consider matching probabilities further in Appendix D.

6 Though, in the smooth ambiguity model, *μ* is a subjective probability, this does not imply that, in the eyes of the model, the agent treats the composition of the first and second-order probabilities as a two-stage lottery. For further discussion in the context of our design, see footnote 11.

7 Subjects undertook an initial practise and some further tasks not relevant to the current paper involving bags of three colours. In this paper, we make no further mention of the irrelevant tasks.

8 In a pilot experiment (*n* = 48), we found that the inferred attitudes to risk and ambiguity did not depend significantly on the order of the tasks.

9 Bags were not described as “risky” or “ambiguous” to subjects. Instead, the white bags were identified numerically and the black bag referred to by that name.

10 For the parenthesised part of this claim, it is important that the black bag did in fact contain some balls of each number. A feature of the procedures explained in Sect. 3.4 is that not only was this true but subjects also had enough information to see its truth.

11 To see the rationale for this interpretation, recall that in the smooth ambiguity model μ is a subjective second-order probability, capturing beliefs about uncertain first-order probabilities (which, in the current context, are values of *p*_*i*_). Ambiguity aversion over first-order events in the smooth model is equivalent to the agent behaving in a more risk averse manner over the domain of second-order events than over the domain of lottery events: as Klibanoff et al. (2005), pp. 1852) put it, an ambiguity-averse agent (in the model) behaves as if the second-order probability is a less reliable guess than a lottery probability. For example, suppose a smooth ambiguity subject in our experiment has *μ*(*p*_1_) = 0.4, i.e., reveals a subjective probability of 0.4 for the second-order event that the proportion of orange balls in the ambiguous bag is *p*_1_. Theoretically, this subject being ambiguity-averse is equivalent to him preferring to bet on a lottery paying 1 with probability 0.4 than to bet the same stake on the event {*p*_1_} and also preferring to bet on a lottery paying with probability 0.6 than to bet the same stake on the event ¬{*p*_1_} Relatedly, formal results discussed in Sect. 2.4 of Klibanoff et al. (2012) show that ambiguity of a first order event *E* (e.g., that an orange ball is drawn from the ambiguous bag) implies that second-order events concerning the probability of *E* (e.g., the probability of an orange ball being drawn is $p_{1}$) are treated as ambiguous, in that the probability of a such an event cannot be calibrated to a lottery probability. Thus, the expected utility evaluation of *second order* acts in the smooth model does not mean that the agent treats these acts as based on unambiguous events.

12 Our approach obtains ambiguity premia in a particular way, based on certainty equivalents. In Appendix D, we demonstrate the feasibility of an alternative approach to calculating ambiguity premia, using the smooth ambiguity model and elicited matching probabilities in an otherwise similar set-up to our own. In the alternative method, unlike in our method, both the certainty equivalents *X* and *Y* would be constructed, rather than directly observed. Construction of *X* is unavoidable as it represents the hypothetical agent; but we see it as a useful feature of our approach that it uses a directly observed *Y*.

13 It is possible to interpret this environment as akin to one of model uncertainty (Marinacci 2015).

14 This “random lottery incentive scheme” is a standard experimental procedure which prevents confounding income effects between tasks which might arise if more than one task was paid. See, for example, Cubitt et al. (1998), Bardsley et al. (2010), Sect. 6.5) and Azrieli et al. (2016) for general discussion. Oechssler and Roomets (2014), Bade (2015) and Baillon et al. (2015) show that an ambiguity sensitive subject could in theory have an incentive to misrepresent their preferences, interpreted as those that would apply to choices among ambiguous acts faced in isolation, if instead they face those tasks in a random lottery incentive scheme. Their argument crucially assumes that the subject views the entire experiment as a single decision problem and, in this respect, is similar to Holt’s (1986)) demonstration that the random lottery incentive system could distort reported risk preferences if subjects violate expected utility theory in the face of objective risk. Partly in view of this, it is an open question whether the crucial assumption holds in practice. In the case of risk, Starmer and Sugden (1991), Beattie and Loomes (1997), Cubitt et al. (1998) and Hey and Lee (2005) tested for the effect conjectured by Holt but found little evidence for it. A similar conclusion is reached by Oechssler et al. (2016) in the context of ambiguity. They find very little evidence of subjects “hedging” across the underlying tasks even when the opportunity to eliminate ambiguity by doing so is made very obvious.

15 In particular, subjects with a CRRA higher (lower) than (minus) 0.03 were classified as being risk averse (seeking). The remaining subjects were classified as being risk neutral.

16 In particular, subjects with a CAAA higher (lower) than (minus) 0.03 were classified as being ambiguity-averse (seeking). The remaining subjects were classified as being ambiguity-neutral.

17 Recall that we elicit CEs for a bet on orange being drawn from the ambiguous bag and also for blue.
